# Association between Active Transportation and Public Transport with an Objectively Measured Meeting of Moderate-to-Vigorous Physical Activity and Daily Steps Guidelines in Adults by Sex from Eight Latin American Countries

**DOI:** 10.3390/ijerph182111553

**Published:** 2021-11-03

**Authors:** Diego Herreros-Irarrázabal, Juan Guzmán-Habinger, Sandra Mahecha Matsudo, Irina Kovalskys, Georgina Gómez, Attilio Rigotti, Lilia Yadira Cortés, Martha Cecilia Yépez García, Rossina G. Pareja, Marianella Herrera-Cuenca, Claudio Farías-Valenzuela, Adilson Marques, Ana Carolina B. Leme, Mauro Fisberg, Clemens Drenowatz, Gerson Ferrari

**Affiliations:** 1 Sports Medicine and Physical Activity Specialty, Science Faculty, Universidad Mayor, Santiago 8580745, Chile; dherrerosi@uft.edu (D.H.-I.); juan.guzmanh@mayor.cl (J.G.-H.); sandra.mahecha@umayor.cl (S.M.M.); 2 Centro de Investigación en Medicina Deporte Ejercicio y Salud—Clínica MEDS, Santiago 7550000, Chile; 3 Nutrition Career, Faculty of Medical Sciences, Pontificia Universidad Católica Argentina, Buenos Aires C1107AAZ, Argentina; ikovalskys@gmail.com; 4 Department of Biochemistry, School of Medicine, Universidad de Costa Rica, San José 11501-2060, Costa Rica; georgina.gomez@ucr.ac.cr; 5 Department of Nutrition, Diabetes and Metabolism, School of Medicine, Pontificia Universidad Católica, Santiago 8330024, Chile; arigotti@med.puc.cl; 6 Department of Nutrition and Biochemistry, Pontificia Universidad Javeriana, Bogotá 110231, Colombia; ycortes@javeriana.edu.co; 7 Colegio de Ciencias de la Salud, Universidad San Francisco de Quito, Quito 17-1200-841, Ecuador; myepez@usfq.edu.ec; 8 Instituto de Investigación Nutricional, Lima 15026, Peru; rpareja@iin.sld.pe; 9 Centro de Estudios del Desarrollo, Universidad Central de Venezuela (CENDES-UCV)/Fundación Bengoa, Caracas 1053, Venezuela; manyma@gmail.com; 10 Instituto del Deporte, Universidad de las Américas, Santiago 9170022, Chile; cfaria46@edu.udla.cl; 11 Department of Didactics of Musical, Plastic and Corporal Expression, Faculty of Education, University of Granada, 18071 Granada, Spain; 12 CIPER, Faculdade de Motricidade Humana, Universidade de Lisboa, 1499-002 Lisbon, Portugal; amarques@fmh.ulisboa.pt; 13 ISAMB, Universidade de Lisboa, 1649-028 Lisbon, Portugal; 14 Centro de Excelencia em Nutrição e Dificuldades Alimentaes (CENDA), Instituto Pensi, Fundação José Luiz Egydio Setubal, Hospital Infantil Sabará, São Paulo 01228-200, Brazil; acarol.leme@gmail.com (A.C.B.L.); mauro.fisberg@gmail.com (M.F.); 15 Family Relations and Applied Nutrition, University of Guelph, Guelph, ON N1G 2W1, Canada; 16 Department of Nutrition, School of Public Health, University of São Paulo, São Paulo 01246-904, Brazil; 17 Departamento de Pediatria, Universidade Federal de São Paulo, São Paulo 04023-061, Brazil; 18 Division of Sport, Physical Activity and Health, University of Education Upper Austria, 4020 Linz, Austria; clemens.drenowatz@ph-ooe.at; 19 Escuela de Ciencias de la Actividad Física, el Deporte y la Salud, Universidad de Santiago de Chile (USACH), Santiago 7500618, Chile

**Keywords:** physical activity, active transportation, public transport, public health, steps per day

## Abstract

This study aimed to examine the associations between active transportation and public transport and the objectively measured meeting of moderate-to-vigorous physical activity (MVPA) and steps per day guidelines in adults by sex from eight Latin American countries. As part of the Latin American Study of Nutrition and Health (ELANS), data were collected from 2524 participants aged 18–65 years. MVPA and steps per day were evaluated using Actigraph GT3X accelerometers. The mode of transportation, its frequency and duration were collected using a self-reported questionnaire. The average time dedicated to active transportation was 12.8 min/day in men (IQR: 2.8–30.0) and 12.9 min/day in women (IQR: 4.3–25.7). A logistic regression analysis was conducted, showing that active transportation (≥10 min) was associated with higher odds of meeting MVPA guidelines (men: OR: 2.01; 95%CI: 1.58–2.54; women: OR: 1.57; 95%CI: 1.25–1.96). These results show a greater association when considering active transportation plus public transport (men: OR: 2.98; 95%CI: 2.31–3.91; women: OR: 1.82; 95%CI: 1.45–2.29). Active transportation plus public transport was positively associated with meeting steps per day guidelines only in men (OR: 1.55; 95%CI: 1.15–2.10). This study supports the suggestion that active transportation plus public transport is significantly associated with meeting the MVPA and daily steps recommendations.

## 1. Introduction

It is known that the current physical activity levels are the lowest in history [1]. This decline in physical activity is a fundamental part of the high prevalence of chronic non-communicable diseases [1]. It is estimated that worldwide, 28 percent of those over 18 years of age do not meet the recommendations of at least 150–300 min of moderate-intensity physical activity or 75–150 min of vigorous-intensity physical activity per week, or an equivalent combination [2]. In Latin America, the decline in physical activity is especially relevant, because it is the area where the highest levels of physical inactivity have been reported, with more than 39.1 percent reporting physical inactivity [3]. In addition to the moderate-to-vigorous physical activity (MVPA) recommendations, an important factor to consider is the number of steps per day. In fact, walking a total of 7000 steps per day is associated with a lower risk of all-cause mortality [4]. Another variable which impacted the development of physical activity levels is private transport, which contributed to physical inactivity and the reduction in the number of steps per day taken by the population. In contrast, active transportation (i.e., walking or cycling) has been suggested as an efficient and economical way to increase physical activity levels. In addition, this type of transport has also been associated with several benefits. For example, it has been associated with positive changes in total physical activity [5] and multiple health benefits [6], mainly associated with a decrease in cardiovascular risk [7].

It is, however, important to consider the methods that have been used to measure physical activity and the number of steps per day, because there are very large variations in the precision of these methods. Among the different methods used to measure physical activity, objective measurements provide more accurate information than subjective measurements [8]. Most publications related to physical activity in Latin America, nevertheless, are based on self-report methods [9,10], which have a significant reporting bias and showed low correlations between subjective and objective physical activity measurements [11]. Conversely, accelerometers require more time and experience in their correct use, in addition to being less economically affordable, even though they provide more accurate and reliable data [11]. Regarding the count of the number of steps per day, current studies have shown that accelerometers provide valid information [12].

Currently, Latin America is one of the most urbanised regions in the world, with about 80 percent of its population living in cities [13]. Urbanisation has generated an increase in mobilisation by motorised vehicles. Accordingly, it has become a fundamental pillar to evaluate the role of active transportation and public transport in the total amount of MVPA and the total number of steps per day, as previous studies have shown a significant decrease in cardiovascular risk with active transportation compared to passive transportation [14]. Therefore, this study aims to examine the associations between active transportation and public transport and meeting MVPA and steps per day guidelines in adults by sex in eight Latin American countries.

## 2. Materials and Methods

### 2.1. Study Design and Participants

The Latin American Study of Nutrition and Health (Estudio Latinoamericano de Nutrición y Salud; ELANS) was a cross-sectional study conducted in eight countries: Argentina, Brazil, Chile, Colombia, Costa Rica, Ecuador, Peru and Venezuela [15,16]. It was an epidemiological study using a standard design with comparable methods across all participating countries. The recruitment of participants was conducted using complex and cluster-stratified multi-stage sample design, with the representation of all regions for each country and a random selection of the main cities in each region, according to the probability proportional to size method. The study focused on urban populations to increase comparability across countries and for feasibility reasons. In total, 92 cities participated in the study and the sampling size required for sufficient precision was calculated with a 95 percent confidence level and a maximum error of 3.5 percent, with a survey design effect of 1.75 and *p* < 0.05, resulting in a required sample size of 9090, based on guidance from the National Center for Health Statistics [17]. Calculations of the minimum sample sizes required per age group, sex, and socioeconomic level were performed for each country. Systematic randomisation was used to determine the range of households within each secondary sampling unit. The selection of the participants belonging to the household was made using the next birthday for 50 percent of the sample, and the last birthday for the other 50 percent. A total of 10,134 (aged 15.0–65.0 years) people were invited to participate in the ELANS study, and 9218 participants (52.2 percent women) between 15 and 65 years of age provided valid data (response rate: 91 percent) and were included in the ELANS study.

Objectively measured physical activity was collected from 40 percent of the sample. To ensure a representative subsample across these dimensions, participants for objective measurements were randomly selected to fill quotas according to sex, age, and socioeconomic level. For logistical and financial reasons, efforts were made to ensure that a range of 23.4–34.2 percent of each sample used the device for five days [18]. The sample used in this article with accelerometer data included 2524 participants aged 18–65 years, representing 27.4 percent of the total ELANS cohort (*n* = 9218). Details have been published elsewhere [18]. We excluded participants ≤ 17 years of age from the analyses because this specific age (15–17 years) was not considered in the sample weight. Adolescents may have different physical activity behaviours compared to adults [19]. Furthermore, physical activity guidelines for adolescents differ from guidelines for adults [20].

Data was collected via two household visits. During the first visit, a subsample of the designated respondents received instructions regarding using the accelerometer, along with a diary to be filled out for seven consecutive days. For the participants who were given accelerometers, the second visit occurred eight days after the first contact. The second visit included the administration of the questionnaire and the retrieval of the accelerometer and diary. There were no significant differences between the participants wearing accelerometers and those who did not by sex (*p* = 0.937), socioeconomic level (*p* = 0.501) or educational level (*p* = 0.235). Participants wearing accelerometers were slightly older (*p* = 0.018) [18].

The ELANS protocol was approved by the Institutional Review Board (#20140605) and registered at ClinicalTrials.gov (#NCT02226627). All the study sites followed the common protocol, with all study personnel undergoing training and certification for data collection. All participants provided informed consent/assent for participation in the ELANS study. The data were collected from September 2014 to February 2015. More information on the ELANS is available elsewhere [15].

### 2.2. Accelerometry Assessment

The Actigraph GT3X accelerometer (Actigraph, Ft. Walton Beach, FL, USA) was used to objectively monitor MVPA and steps per day. The accelerometer was worn for seven days on an elasticated belt at hip level on the right mid-axillary line. Participants were requested to wear the device while awake and to remove it when sleeping, showering or swimming for at least seven days and at least 12 h/day. The minimum amount of accelerometer data considered acceptable for analytical purposes was five days (with at least 1 weekend day) and at least 10 h/day of wear time following the removal of sleep time [21,22]. After excluding the nocturnal sleep period, waking non-wear time was defined as any sequence of at least 60 consecutive minutes of zero activity counts.

Data were processed using ActiLife software (V6.0; ActiGraph, Pensacola, FL, USA). Data were collected at a sampling rate of 30 Hz and downloaded in epochs of 60 s [23]. MVPA was defined as time taken to accumulate ≥ 1952 activity counts/min (≥3.0 METs) [24,25,26,27]. The reliability and validity of accelerometers have been documented previously [15,28]. To account for intensity, total physical activity (defined as total MVPA in minutes per week) was weighted by multiplying vigorous physical activity by 2: total physical activity = moderate physical activity (minutes per week) + [2 × vigorous physical activity (minutes per week)]. Participants were categorised as “meeting” or “not meeting” MVPA guidelines if they completed ≥ 150 min/week of MVPA [2,29]. To calculate mean daily step counts, the total number of steps during the valid wear period was divided by the number of valid days that each participant was wearing the accelerometer. Daily step counts measured over 7 days using the ActiGraph GT3X+ was previously shown to correlate (r = 0.86 for men; r = 0.89 for women) well to physical activity energy expenditure as measured by doubly labeled water [30]. We defined those who walked ≥ 7000 steps as meeting the recommendations regarding walking for health, given the impact that it has on all-cause mortality in middle-aged adults [4].

### 2.3. Active Transportation and Public Transport

Measurements were collected according to standardised procedures in each country. Mode of transportation, its frequency and duration were collected using a questionnaire. To investigate the form of transportation, the mode of transport and the amount of time spent on active transportation (walking or cycling) were asked about, as detailed next. Participants were asked about their mode of transport that they generally used to move from one place to another (bus, taxi, subway, train, car and motorcycle). Public transport included traveling by bus, taxi, subway, and train. For this article, we consider participants who selected any of these means of transportation as the first option as part of the group that uses public transport.

Regarding active transportation, we refer to the measures as overall travel rather than commuting. The questions captured overall travel rather than travel specifically undertaken to/from work. Active transportation was evaluated at the second visit using a Spanish language long-form, last 7-day, self-administered version of the International Physical Activity Questionnaire (IPAQ) [31]. The long-form IPAQ (last 7 days) has been validated internationally using CSA accelerometer (model 7164) to assess total physical activity in a variety of contexts (occupational, transport, household, leisure) and at different intensities (moderate, vigorous, walking, cycling) in individuals aged 18–65 years from 12 countries with Spearman’s correlation coefficients ranging from 0.46 to 0.96 [31]. For active transportation, the following questions were asked: (i) “Do you walk or use a bicycle (pedal cycle) for at least 10 min continuously to get to and from places?” (Yes, No); (ii) “In a typical week, how many days do you walk or ride a bicycle for at least 10 min continuously to get to and from places?” and (iii) “How much time do you spend walking or bicycling for travel on a typical day?” These questions were asked separately for walking and cycling. The time spent undertaking active transportation was expressed as min/day. In this study, we classified participants into two categories for active transportation, those who did ≥ 10 min/day and those who did < 10 min/day. Among the participants who completed ≥ 10 min/day, we created a subcategory to determine the impact of the use of public transport.

### 2.4. Sociodemographic Variables

Information about demographics, including age, sex and years of education, was collected using standard questionnaires. Due to the different classification systems used among different countries, three levels of classification based on the national statistics used in each country were used with equivalent characteristics for all countries. Individuals self-reported their sex, age (18–34.9, 35–49.9, and 50–65 years), and education level (basic or lower [low], high school [middle], and university degree [high]). Data on socioeconomic level were divided into three strata (low, medium, high) according to the national indexes used in each country. Full information can be found in an earlier publication [15]. The sample was stratified by sex, age range, education level (low, middle and high) and socioeconomic level (low, middle, and high). Socioeconomic levels were weighted according to the national indices of each country.

### 2.5. Statistical Analysis

Data were weighted considering sociodemographic variables, sex, age and socioeconomic level [20,31,32]. All variables were tested for normal distribution using the Kolmogorov–Smirnov test. Active transportation, MVPA, and steps per day were described using absolute frequencies and median (with interquartile range: IQR) due to the lack of normal distribution. Analyses were carried out according to country, age group, socioeconomic and education level. Due of the significant differences between men and women in levels of MVPA and steps per day [20,33], we conducted analyses separately for men and women.

For descriptive and categorical analyses, the variables were analysed separately and stratified into the following two categories: (1) active transportation: self-reported time who spent < 10 min or ≥10 min of walking and/or cycling for transportation; (2) active transportation plus public transport: who spent < 10 min of active transportation without use of active transport or ≥10 min of active transportation plus use of public transport; (3) meeting MVPA guidelines: < 150 min/week or ≥150 min/week [2]; (4) meeting steps per day: <7000 or ≥7000 [4].

Chi-squared tests were used to examine differences in active transportation, active transportation plus public transport, meeting MVPA guidelines, and meeting steps per day guidelines by sex and sociodemographic variables.

A logistic regression model was used to estimate the odds ratios (OR) and 95 percent confidence intervals (95 percent CI) for the associations of active transportation (also plus public transport) with meeting vs. not meeting (0 = “<150 min/week”, 1 = “≥150 min/week”) MVPA guidelines, and meeting vs. not meeting (0 = “<7000”, 1 = “≥7000”) steps per day guidelines according sex adjusted for country, age, education level and socioeconomic level. A significance level of 5 percent was adopted. Statistical analyses were carried out with the software SPSS v.26 (SPSS Inc., IBM Corp., Armonk, New York, NY, USA).

## 3. Results

The total number of participants included in the study was 2524 (1341 [53.1 percent] women), and the mean age was 38.3 (standard deviation: 13.4) years. Overall, the participants self-reported a median of 12.8 (IQR: 3.6–30.0) min/day of active transportation. The medians of MVPA and steps per day evaluated by accelerometer were 28.3 (IQR: 16.4–46.4) min/day and 9706.5 (IQR: 6747.8–14,018.1), respectively. The median (IQR) duration of time spent in active transportation, MVPA and steps per day by country, age and sociodemographic characteristics among participants are shown in Table 1. The countries with the highest levels of active transportation for men and women were Ecuador and Costa Rica. In contrast, the country with the lowest level was Venezuela. A marked difference was observed in the levels of active transportation between men and women in Chile and Colombia, where men participated in between 30 and 33 percent more active transportation than women. In Peru and Argentina, women participated in between 21 and 25 percent more active transportation than men. There was a marked difference in the levels of MVPA between men (35.2 min/day) and women (23.7 min/day). This difference by sex was less noticeable for steps per day (10,291 and 9155 steps/day, respectively). Those in the high socioeconomic status group reported more time partaking in active transportation. Still, the accelerometer measurement showed less MVPA and fewer steps per day.

Regarding the proportion of active transportation (≥10 min), active transportation plus public transport, meeting MVPA and steps per day guidelines by sociodemographic characteristics, these data are presented in Table 2. A very similar proportion of men and women partake in at least 10 min of active transport per day (57.5 and 57.6 percent). However, within this group, more women also used public transport as their main mode of transport (32.0 percent and 37.3 percent). The country with the highest proportion of people who perform active transportation or active transportation and use public transport was Ecuador in both men and women. There was a trend according to socioeconomic group in men and women, with the high socioeconomic group reporting the highest proportion of active transportation (61.7 and 58.4 percent). Meeting the MVPA was markedly higher in men than in women (59.0 and 38.5 percent). There was a significant difference by age group. The highest proportion that met the recommendation was men aged 18–35 years, and the lowest was women aged 50–65 years (63.8 and 35.0 percent). A difference was also found according to socioeconomic level, with the lower income group having a lower probability of meeting the MVPA recommendation. Regarding the educational level, it was observed that the group of women in the intermediate level had both the highest levels of active transportation plus public transport (42.0 percent) and the highest proportion of meeting the MVPA recommendation among women (40,9 percent). Regarding meeting the recommendation of steps per day, a difference was observed by sex (75.4 and 69.9 percent), especially in the 18–35 age group (76.4 and 68.3 percent), in the low socioeconomic level (78.3 and 69.5 percent) and in the low educational level (74.0 and 65.7 percent).

The results of the logistic regression analysis describing the association between active transportation, and active transportation plus public transport with meeting vs. not meeting the MVPA and steps per day guidelines regardless of country, age, education level and socioeconomic level are presented in Table 3. Active transportation (men: OR: 2.01; 95 percent; CI: 1.58–2.54; women: OR: 1.57; 95 percent; CI: 1.25–1.96) and active transportation plus public transport (men: OR: 2.98; 95 percent; CI: 2.31–3.91; women: OR: 1.82; 95 percent; CI: 1.45–2.29) were associated with higher odds of meeting MVPA guidelines. Furthermore, active transportation plus public transport (OR: 1.55; 95 percent; CI: 1.15–2.10) was positively associated with meeting steps per day guidelines only in men. Active transportation and active transportation plus public transport were not associated with meeting steps per day guidelines in women.

## 4. Discussion

The present study examined the associations between active transportation and public transport and meeting MVPA and steps per day guidelines among adults by sex from eight Latin American countries. The results indicate that active transportation (≥10 min) plus public transport is associated with a greater likelihood of meeting the MVPA (both men and women) and steps per day guidelines (only in men) compared to participants participating only in active transportation.

Although the association between active transport (≥10 min/day) plus public transport and MVPA guidelines is significant in both men and women, a stronger association is seen in the male population than in the female population. This difference could be related to the intensity of physical activity performed by men, which would be higher when compared to women. This, however, warrants further investigation. Conversely, the non-existence of a significant relationship between active transportation plus public transport (≥10 min) with steps per day guidelines could be related to the different types of active transportation (walking v/s bicycle). Undoubtedly, there are interesting variables to clarify in future research. In line with our findings, previous studies [34,35,36] from high-income countries reported positive associations between active transportation and physical activity; therefore, transport and health policies are indivisible. Some Latin American countries have implemented programs to positively influence physical activity. Bus Rapid Transit (BRT) systems, car use restriction measures, “*Ciclovía*” programs, and the construction of cycle paths [32,37,38,39]. For example, BRT systems have been widely implemented as a cost-effective solution for urban mobility [40], but they also can stimulate the use of active modes of transport and reduce private car use, thus promoting physical activity [41]. Furthermore, one of the policies to increase active transportation for the population may be the development of neighbourhoods and cities favouring this type of transport [32,38]. Our study suggests that the promotion of active transportation along with the use of public transport has the potential to increase total physical activity.

The mode of transportation not only affects human health through physical activity, but we must also consider the environmental impact. It is increasingly recognised that air pollution hurts human health [42]. Rojas-Ruedas et al. studied different scenarios to reduce the use of private transport and increase the use of bicycles and the use of public transport in urban areas. They found that it would not only have a direct impact on health, but would also have an impact on air quality and greenhouse gas emissions [43].

Our study has some limitations. Only urban areas were included in the ELANS, and thus the samples are not nationally representative, even though a large number of citizens in Latin America live in urban areas. The cross-sectional design of the study also precludes the determination of causality, even with careful adjustment for covariates. Active transportation and public transport data were self-reported, which can lead to some bias. The active transportation information is restricted to time spent walking or cycling, and it does not refer to time spent in the usage of public transport. This restricts the analysis to looking at active transportation and not the individual choice of travel mode. It is not possible to examine the association between the use of public transportation and active commuting in this study, but only the association between the first option of public transport and active transportation in general. Cultural differences have been reported in the interpretation of IPAQ, and the lack of precision of IPAQ compared with objective measures of physical activity [11] could decrease the likelihood of significant associations with outcomes. Nonetheless, the strengths of the study include a large sample from several countries, which enables us to assess the relationship between active transportation and accelerometer-measured physical activity in the Latin American population. Accelerometers, such as those used in ELANS, are valid instruments to measure steps and physical activity intensity [18,44,45]. The use of device methods is rare in Latin American countries, where most previous research relied on self-reported instruments [33,46,47]. The promotion and facilitation of active transportation and public transport should be part of international and global strategies for preventing physical inactivity, and it should be mandatory for policy makers and planners to take active transportation facilities into account.

## 5. Conclusions

This study supports the suggestion that active transportation plus the use of public transport is significantly associated with meeting the MVPA and steps per day recommendations in adults from eight Latin America countries. Considering the multiple benefits associated with reducing the levels of private transport in health, society and the environment, promoting and investing in active transportation and public transport should be a priority at the public policy level in Latin America.

## Figures and Tables

**Table 1:** Sociodemographic characteristics (median [25–75]) according to active transportation, moderate-to-vigorous physical activity and steps per day in adults from Latin America.

Variables		Active Transportation (min/day)		Moderate-To-Vigorous Physical Activity (min/day)		Steps per Day	
	*n* (%)	Men	Women	Men	Women	Men	Women
Overall	2524	12.8 (2.8–30.0)	12.9 (4.3–25.7)	35.2 (20.6–56.8)	23.7 (14.0–38.3)	10291.8 (7032.1–14,606.1)	9155.7 (6474.1–13,165.3)
**Country**							
Argentina	271 (10.7)	11.1 (0.0–30.0)	14.0 (4.3–30.0)	28.7 (16.9–47.8)	26.7 (14.6–43.9)	7497.9 (5450.4–9956.8)	7616.7 (5810.5–9566.3)
Brazil	524 (20.8)	10.7 (0.0–30.0)	8.6 (2.9–21.4)	32.6 (18.7–54.8)	22.7 (13.7–38.6)	14,577.8 (10,562.7–18,625.3)	13,324.0 (10,089.6–16,359.5)
Chile	274 (10.9)	17.2 (4.3–30.0)	12.0 (4.3–25.7)	39.0 (25.0–60.8)	31.2 (21.1–44.8)	15,631.3 (12,954.4–18,618.1)	14,111.6 (11,141.6–16,784.0)
Colombia	319 (12.6)	17.1 (6.4–34.0)	11.4 (4.5–23.9)	36.7 (22.5–51.7)	25.3 (12.0–39.4)	7580.6 (5915.3–10,379.0)	7490.8 (5663.5–9844.5)
Costa Rica	247 (9.8)	17.1 (4.3–60.0)	17.1 (8.6–40.0)	33.4 (18.6–54.2)	20.6 (9.9–35.2)	7800.8 (5555.3–10,324.6)	6747.0 (4945.9–8544.7)
Ecuador	249 (9.9)	17.1 (8.6–30.0)	16.1 (8.6–30.0)	42.4 (24.1–64.8)	23.4 (15.5–37.8)	8753.8 (6365.4–11,189.2)	6710.7 (5606.8–9130.1)
Peru	302 (12.0)	12.9 (5.7–30.0)	17.1 (6.4–30.0)	38.9 (22.6–60.3)	24.7 (15.5–39.2)	8562.6 (6037.3–11,322.6)	7415.5 (5947.5–9478.0)
Venezuela	338 (13.4)	4.3 (0.0–15.0)	6.1 (0.0–15.0)	35.2 (17.0–55.4)	19.3 (12.2–29.9)	12,823.1 (9710.3–16,604.8)	11,285.7 (7539.0–14,877.3)
**Age Group**							
18–34	1134 (44.9)	12.9 (2.9–30.0)	14.3 (5.7–30.0)	36.6 (23.3–58.1)	24.4 (15.6–37.9)	10,342.8 (7100.2–14,588.7)	8827.3 (6350.1–12,657,2)
35–49	782 (31.0)	12.9 (0.0–30.0)	10.7 (4.3–25.7)	35.0 (18.8–59.1)	25.4 (13.8–39.7)	10,488.6 (7122.3–14,877.6)	9147.0 (6647.8–13,361.9)
50–65	608 (24.1)	14.3 (4.3–32.1)	11.1 (2.9–25.7)	30.8 (17.8–52.1)	21.3 (11.3–36.1)	9899.2 (6503.0–14,292.5)	9611.2 (6473.5–13,711.3)
**Socioeconomic Level**							
Low	1287 (51.0)	12.6 (1.5–30.0)	12.9 (4.3–25.0)	37.0 (20.8–61.5)	23.5 (13.8–37.3)	10,790.6 (7544.9–15,125.8)	8964.1 (6444.1–13,141.6)
Middle	980 (38.8)	12.8 (2.8–30.0)	12.0 (4.3–25.7)	34.3 (20.6–54.5)	24.9 (14.2–40.1)	10,128.8 (6911.6–14,482.4)	9523.7 (6510.7–13,358.7)
High	257 (10.2)	14.3 (5.7–33.7)	15.0 (5.0–30.0)	31.3 (18.3–47.9)	22.4 (14.0–46.3)	8869.3 (6158.6–12,895.9)	8828.9 (6417.3–12,550.0)
**Education Level**							
Low	1457 (57.7)	12.9 (2.9–32.9)	12.9 (4.3–25.7)	34.8 (19.2–59.7)	22.6 (12.9–37.5)	10,222.3 (6903.7–14,869.9)	8745.3 (6173.8–12,590.6)
Middle	794 (31.5)	11.4 (1.7–26.4)	12.6 (4.3–28.6)	35.2 (21.5–55.3)	26.8 (16.7–41.3)	10,291.8 (7264.3–14,277.5)	10,098.2 (7217.1–13,672.8)
High	273 (10.8)	14.3 (1.2–30.0)	11.4 (2.1–30.0)	35.9 (21.8–52.4)	23.0 (14.0–35.1)	10,591.1 (7067.5–14,528.5)	9084.3 (6882.8–13,627.4)

**Table 2:** Descriptive characteristics (*n* (%)) of participants by active transportation (also plus public transport), meeting moderate-to-vigorous physical activity and steps per day guidelines.

Variables	Active (≥10 min) Transportation (%) (*n* = 1453)		Active (≥10 min) Transportation (%) plus Public Transport (*n* = 879)		Meeting Moderate-To-Vigorous Physical Activity Guidelines (*n* = 1213) ^1^		Meeting Steps per Day Guidelines (*n* = 1828) ^2^	
	Men	Women	Men	Women	Men	Women	Men	Women
Overall	680 (57.5)	773 (57.6)	379 (32.0)	500 (37.3) *	698 (59.0)	515 (38.5) *	892 (75.4)	936 (69.9) *
Country								
Argentina	62 (54.4)	91 (58.0)	29 (25.4)	60 (38.2) *	56 (49.1)	69 (44.5)	64 (56.1)	92 (59.4)
Brazil	123 (53.2)	143 (48.8)	61 (26.4)	85 (29.0)	125 (54.1)	103 (35.2) *	211 (91.3)	275 (93.9)
Chile	83 (65.4)	89 (60.5)	45 (35.4)	61 (41.5)	83 (65.4)	79 (53.7) *	126 (99.2)	141 (95.9)
Colombia	110 (69.2)	91 (56.9) *	51 (32.1)	44 (27.5)	100 (62.9)	68 (42.5) *	91 (57.2)	90 (56.3)
Costa Rica	72 (61.5)	89 (68.5)	33 (28.2)	54 (41.5) *	63 (53.8)	44 (33.8) *	71 (60.7)	56 (43.1) *
Ecuador	88 (70.4)	92 (74.2)	68 (54.4)	67 (54.0)	84 (67.2)	50 (40.3) *	83 (66.4)	58 (46.8) *
Peru	84 (59.2)	109 (68.1)	51 (35.9)	80 (50.0) *	90 (63.4)	60 (37.5) *	98 (69.0)	91 (56.9) *
Venezuela	58 (34.5)	69 (40.6)	41 (24.4)	49 (28.8)	97 (57.7)	42 (24.7) *	148 (88.1)	133 (78.2) *
Age Group								
18–34	328 (57.3)	356 (63.3) *	199 (34.8)	245 (43.6) *	365 (63.8)	217 (38.6) *	437 (76.4)	302 (68.3) *
35–49	198 (55.8)	226 (52.9)	101 (28.5)	126 (29.5)	204 (57.5)	175 (41.1) *	271 (76.3)	302 (70.9)
50–65	154 (60.2)	191 (54.3)	79 (30.9)	129 (36.6)	129 (50.4)	123 (35.0) *	184 (71.9)	250 (71.2)
Socioeconomic Level								
Low	337 (56.4)	396 (57.5)	198 (33.1)	257 (37.1)	363 (60.7)	253 (36.7) *	468 (78.3)	479 (69.5) *
Middle	269 (57.8)	297 (57.7)	145 (31.2)	191 (37.1)	268 (57.6)	210 (40.9) *	346 (74.4)	362 (70.4)
High	74 (61.7)	80 (58.4)	36 (30.0)	52 (38.0)	67 (55.8)	52 (38.2) *	78 (65.0)	95 (69.9)
Education Level								
Low	398 (58.7)	461 (59.2)	224 (33.0)	274 (35.3)	389 (57.4)	279 (35.9) *	502 (74.0)	511 (65.7) *
Middle	204 (54.1)	233 (55.9)	115 (30.5)	175 (42.0) *	227 (60.2)	182 (43.6) *	292 (77.5)	318 (76.3)
High	78 (60.9)	79 (54.5)	40 (31.3)	51 (35.2)	82 (64.1)	54 (37.5) *	98 (76.6)	107 (74.3)

* Chi-square Tests were used for difference between sex (*p* < 0.05); **^1^** defined as ≥150 min/week; **^2^** defined as ≥7000 steps per day.

**Table 3:** Logistic regression (OR (95%CI)) for active transportation (also plus public transport) with meeting moderate-to-vigorous physical activity and steps per day guidelines.

Variables	Meeting Moderate-To-Vigorous Physical Activity Guidelines ^1^		Meeting Steps per Day Guidelines ^2^	
	OR (95%CI)	*p*-Value	OR (95%CI)	*p*-Value
Men				
Active transportation		<0.001 *		0.321
<10 min	1		1	
≥10 min	2.01 (1.58–2.54)		1.14 (0.87–1.49)	
Active transportation plus public transport		<0.001 *		0.004 *
<10 min	1		1	
≥10 min	2.98 (2.31–3.91)		1.55 (1.15–2.10)	
Women				
Active transportation		<0.001 *		0.332
<10 min	1		1	
≥10 min	1.57 (1.25–196)		0.12 (0.88–1.42)	
Active transportation plus public transport		<0.001 *		0.062
<10 min	1		1	
≥10 min	1.82 (1.45–2.29)		1.26 (0.98–1.61)	

Logistic regression model adjusted for country, age, education level and socioeconomic level; OR: odds ratio; CI: confidence interval; **^1^** 0 = <150 min/week; 1 = ≥150 min/week; **^2^** 0 = <7000 steps per day; 1 = ≥7000 steps per day; * statistical significance (*p* < 0.05).

## Data Availability

The datasets generated and/or analysed during the current study are not publicly available due the terms of consent/assent to which the participants agreed but are available from the corresponding author on reasonable request. Please contact the corresponding author to discuss availability of data and materials.
